# Design and Experimental Validation of a Piezoelectrically Controlled Micro-Newton Cold-Gas Thruster Head

**DOI:** 10.3390/mi17080876

**Published:** 2026-07-23

**Authors:** Xiaocheng Zhu, Oleksii Cherkun, Jie Xu, Zhan Hu, Bin Wang, Haiying Hu, Zhiming Cai, Bin Guo

**Affiliations:** 1 School of Materials Science and Engineering, Harbin Institute of Technology, Harbin 150001, China; zhuxc@microsate.ac.cn (X.Z.);; 2 Innovation Academy for Microsatellites, Chinese Academy of Sciences, Shanghai 201304, China; 3 Key Laboratory for Satellite Digitalization Technology, Chinese Academy of Sciences, Shanghai 201210, China

**Keywords:** cold-gas microthruster, integrated thruster head, micro-nozzle, piezoelectric actuator, DSMC, rarefied gas flow, mass-flow calibration, space gravitational-wave detection

## Abstract

Micro-Newton cold-gas thrusters are promising actuators for precision space missions, but their performance is strongly influenced by the integrated head architecture. This study presents the design, fabrication, and experimental validation of a piezoelectrically controlled cold-gas microthruster head for space-based gravitational-wave detection missions. The proposed head integrates a cone-needle throttle, a micro-orifice interface, and a downstream micro-nozzle, thereby converting actuator displacement into a regulated mass flow and ultimately into thrust. One-dimensional theory was first used for preliminary sizing, and Direct Simulation Monte Carlo (DSMC) analysis of the complete throttle-nozzle geometry was then applied to determine the final design parameters under rarefied-flow conditions. The selected design uses a throat radius of 29 μm and a needle half-angle of 10 degrees. Following fabrication and structural characterization, the integrated device was validated through mass-flow calibration and vacuum thrust testing. The experimental results show that the pressure-decay-based calibration provides a consistent mapping between actuation command, calibrated flow rate, and thrust output. The measured flow–thrust relation preserves the high linearity predicted by simulation, while the experimentally evaluated specific impulse meets the specified design target over the tested range. In addition, thrust-resolution testing at a baseline thrust of approximately 98.4 micro-Newton demonstrates a minimum resolvable step of 50 nano-Newton, and the measured thrust-noise amplitude spectral density remains below 0.07 micro-Newton/sqrt(Hz) over the 10 mHz–1 Hz band for the tested thrust levels. These results support the feasibility of the proposed integrated cold-gas microthruster head and its device-level validation approach for future space-based gravitational-wave detection applications.

## 1. Introduction

### 1.1. Space Gravitational-Wave Mission Requirements for Micro-Newton Cold-Gas Thrusters

Space gravitational-wave detection missions extend gravitational-wave observation from ground-accessible high-frequency bands to low-frequency bands accessible only from space. The European Space Agency’s LISA mission, together with China’s Taiji and TianQin programs, aims to detect extremely weak low-frequency gravitational-wave signals in space [1,2,3,4,5]. As an in-orbit technology precursor to LISA, LISA Pathfinder completed a key demonstration of the drag-free control and free-fall performance required for space gravitational-wave detection [6,7]. To keep the test masses as close as possible to pure free fall, these missions rely on drag-free control to suppress non-conservative force disturbances, including solar pressure, rarefied atmospheric drag, and thermal-radiation recoil. High-precision micropropulsion has therefore become a key actuation technology for space gravitational-wave detection platforms.

Among the available micropropulsion approaches, cold-gas microthrusters offer several advantages, including a clean and non-contaminating working medium, continuously adjustable thrust, a relatively simple control chain, and compatibility with high-precision inertial-measurement payloads. These advantages were demonstrated in orbit by LISA Pathfinder, whose drag-free and attitude-control system employed cold-gas micropropulsion actuators. Dedicated in-flight characterization reported stable science-mode operation with an average thruster-noise level of approximately $0.17\;\mu N/\sqrt{\mathrm{Hz}}$, and the measured thruster performance was shown to be compatible with the noise budget required for the upcoming LISA gravitational-wave observatory [8,9]. Accordingly, the current LISA definition study specifies cold-gas micro-Newton thrusters as the baseline actuators for drag-free attitude control [10]. For applications requiring micro-Newton thrust, low noise, and long-duration stable output, cold-gas microthrusters can provide a calibratable chain linking throttle control, mass flow, and thrust output, which makes them a promising route for space gravitational-wave micropropulsion [11,12,13].

The central development challenge is that several mission-level indicators must be satisfied simultaneously rather than independently. LISA-oriented propulsion and thrust-stand studies have specified micro-Newton continuous thrust, sub-0.1 μN resolution, and thrust noise below about $0.1\;\mu N/\sqrt{\mathrm{Hz}}$ in the science band [14,15]. For a cold-gas device, these requirements simultaneously constrain the minimum controllable valve opening, gas-path dead volume, nozzle expansion efficiency, actuator dynamics, and flow/thrust calibration accuracy. Recent Taiji-oriented cold-gas development has further demonstrated wide thrust authority, 0.05 μN resolution, low noise, and sub-second response, but the reported response and noise results also show that nozzle design, valve dynamics, flow sensing, and thrust measurement must be treated as a coupled problem [16]. Representative state-of-the-art requirements and results are summarized in Table 1.

### 1.2. State of the Art in Micro-Newton Cold-Gas Micropropulsion Technology

Research on micro-Newton cold-gas micropropulsion has evolved from compact micro-electro-mechanical systems (MEMS) demonstrators toward mission-oriented proportional thrusters with low noise, wide authority, and embedded sensing. Early international work integrated microfabricated nozzles, valves, filters, and flow channels for spacecraft fine pointing [17]. Recent reviews for space gravitational-wave detection identify cold gas as a mature and contamination-free micropropulsion route, while emphasizing that performance is governed by the coupled design of the valve, nozzle, flow sensor, and control electronics [18]. Taiji- and TianQin-oriented studies have further reported wide-range proportional valves [16,19], MEMS or silicon nozzles [20], low-noise flow sensors [11,12], and integrated cold-gas prototypes with embedded sensing and thrust measurement [13].

Recent technical progress can be grouped into three areas. First, micron-scale nozzles and throttle gaps often operate in the transition regime, so continuum sizing must be corrected by DSMC or hybrid continuum–DSMC methods to capture wall slip, gas–surface accommodation, finite-gap throttling, and plume nonequilibrium [21,22,23,24,25,26,27,28]. Second, manufacturing must form micron-scale throats and valve seats while preserving sealing repeatability, alignment, and dimensional traceability; recent silicon nozzles and piezoelectric proportional valves demonstrate sub-0.1 μN or sub-0.1 μg/s resolution, but still require careful control of sealing conditions, zero offset, and assembly tolerance [19,20,29,30]. Third, micromachined thermal sensors and integrated multi-sensor thrusters provide the calibration bridge from actuator command to mass flow and thrust [11,12,13,31]. These studies indicate a shift from isolated component optimization toward a closed design–simulation–fabrication–calibration chain.

### 1.3. Research Focus of This Paper

This work develops and validates a micro-Newton cold-gas thruster head intended for the drag-free control requirements of space gravitational-wave detection missions. The work focuses on the device-level realization of the cold-gas microthruster rather than the complete spacecraft control loop. In particular, it investigates the design, rarefied-flow simulation, fabrication, integration, and ground validation of the piezoelectrically controlled head.

The target design indicators for the thruster head include a thrust range of 0–200 μN, thrust resolution better than 0.1 μN, a thrust-noise target below $0.1\;\mu N/\sqrt{\mathrm{Hz}}$ in the 10 mHz–1 Hz band, specific impulse greater than 50 s for F≥10 μN, and response time shorter than 150 ms. The 0.1 mHz–10 mHz range is treated only as a low-frequency envelope check based on a power spectral density (PSD) slope of −40 dB/dec. To address these targets, this study covers the cone-needle throttle and micro-nozzle design, DSMC-based correction of the complete micro-scale flow path, fabrication and dimensional inspection of the key components, and integrated verification through flow calibration and micro-thrust measurement.These target indicators and their design implications are summarized in Table 2.

Among these target design indicators, the present study experimentally verifies the flow–thrust relation, specific impulse, thrust resolution, and thrust-noise performance of the fabricated thruster head, while response time is retained as a design-side constraint for the integrated head architecture.

## 2. Theoretical Calculation and Simulation Optimization

### 2.1. Conceptual Design and Theoretical Calculation

#### 2.1.1. Conceptual Design

**Figure 1:**
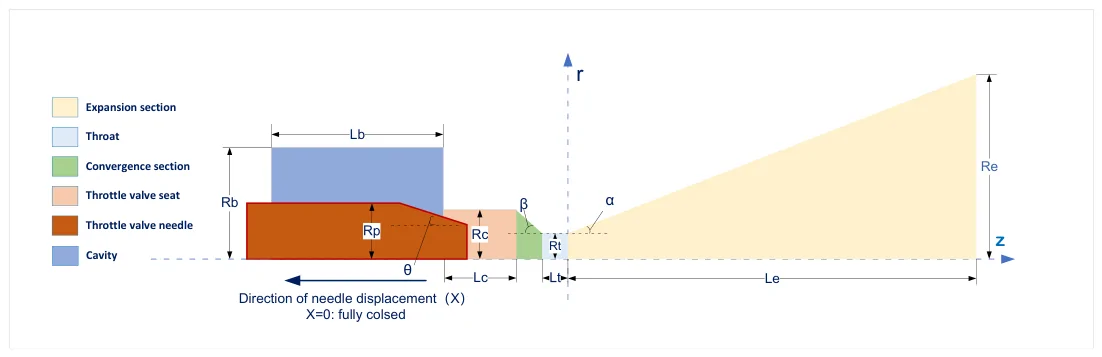
Conceptual design of the integrated throttle-nozzle cold-gas microthruster head, showing the shortened control path from the cone-needle metering interface to the converging section, throat, and expansion section.

The microthruster head is designed around an integrated throttle-nozzle valve architecture rather than as a separate proportional valve connected to a downstream nozzle. As shown in Figure 1, the piezoelectric actuator changes the axial position of the cone needle, the cone-needle/orifice gap sets the effective throttling area, and the gas subsequently passes through the converging section, throat, and expansion section within the same compact structure. This arrangement forms a short, direct transfer path from throttling control to momentum conversion.

This integrated concept is intended to improve device-level control precision and response speed. By placing the throttling interface, converging section, throat, and expansion passage in one continuous micro-scale flow path, the design reduces intermediate dead volume, alignment-sensitive interfaces, and additional pneumatic lag.

#### 2.1.2. Theoretical Calculation

Based on one-dimensional isentropic nozzle theory and the geometric relationship of the cone-needle/orifice throttling interface, a preliminary static relation between cone-needle displacement and thrust is derived [24,26]. This preliminary model is used only for initial geometric sizing before complete-geometry DSMC correction. Nitrogen is treated as an ideal gas, the nozzle flow is assumed to be steady, one-dimensional, and isentropic, and the ambient pressure is approximated as vacuum. Finite-gap and wall-collision losses, entrance-contraction loss, rarefaction, and local nonequilibrium are not included in this preliminary model and are subsequently evaluated using DSMC simulation.

The chamber pressure and temperature are set to $p_{c}=1\;\mathrm{bar}$ and $T_{c}=298.15\;K$, respectively. A preliminary design thrust of $F_{d}=250\;\mu N$ is used to retain margin above the nominal 200 μN upper requirement. The initial nozzle-sizing parameters include an expansion ratio $\epsilon =A_{e}/A_{t}=25$, where $A_{e}$ is the nozzle exit area and $A_{t}$ is the throat area, an expansion half-angle $\alpha_{e}=15^{{}^{\circ}}$, and a contraction half-angle $\alpha_{c}=30^{{}^{\circ}}$. The remaining parameters are a contraction ratio $\lambda_{c}=A_{c}/A_{t}=4$, where $A_{c}$ denotes the inlet area of the converging section, and a needle stroke $z_{\max}=50\;\mu m$.

For the preliminary one-dimensional nozzle sizing, the thrust is written as

(1) $$F=\dot{m}v_{e}+\left(p_{e}-p_{a}\right)A_{e},$$

where *F* is the thrust, $\dot{m}$ is the mass flow rate, $v_{e}$ is the nozzle-exit velocity, $p_{e}$ is the exit pressure, and $p_{a}$ is the ambient pressure. In the one-dimensional isentropic preliminary model, the prescribed expansion ratio determines the exit Mach number $M_{e}$ through

(2) $$\frac{A_{e}}{A_{t}}=\frac{1}{M_{e}}{\left[\frac{2}{\gamma +1}\left(1+\frac{\gamma -1}{2}M_{e}^{2}\right)\right]}^{\frac{\gamma +1}{2(\gamma -1)}},$$

where γ is the specific-heat ratio of nitrogen. The corresponding exit pressure is

(3) $$p_{e}=p_{c}{\left(1+\frac{\gamma -1}{2}M_{e}^{2}\right)}^{-\frac{\gamma }{\gamma -1}}.$$

The thrust coefficient is then estimated by

(4) $$C_{f}=\sqrt{\frac{2\gamma^{2}}{\gamma -1}{\left(\frac{2}{\gamma +1}\right)}^{\frac{\gamma +1}{\gamma -1}}\left[1-{\left(\frac{p_{e}}{p_{c}}\right)}^{\frac{\gamma -1}{\gamma }}\right]}+\frac{p_{e}-p_{a}}{p_{c}}\frac{A_{e}}{A_{t}},$$

where $C_{f}$ is the thrust coefficient. Introducing a preliminary discharge coefficient $C_{d}$, the throat area required by the design thrust is obtained from

(5) $$A_{t}=\frac{F_{d}}{C_{f}C_{d}p_{c}}.$$

After $A_{t}$ is determined, the exit diameter and the axial lengths of the converging and diverging sections are calculated from ϵ, $\lambda_{c}$, $\alpha_{c}$, and $\alpha_{e}$.

The static thrust–control relation can therefore be expressed as a mapping from cone-needle displacement to effective throttling area. For the cone-needle/orifice interface, the small-gap annular area $A_{\mathrm{eff}}$ is approximated as

(6) $$A_{\mathrm{eff}}\left(z,\theta \right)\approx \pi \sin\theta \left(D_{i}z-z^{2}\sin\theta \cos\theta \right),$$

where *z* is the axial displacement of the cone needle, $D_{i}$ is the inlet-orifice characteristic diameter, and θ is the needle half-angle. To make the full-open throttling area consistent with the preliminary throat area, the endpoint constraint $A_{\mathrm{eff}}\left(z_{\max},\theta \right)=A_{t}$ is imposed, giving

(7) $$D_{i}\left(\theta \right)=\frac{A_{t}}{\pi z_{\max}\sin\theta }+z_{\max}\sin\theta \cos\theta .$$

Substituting Equation (7) into Equation (6) gives

(8) $$A_{\mathrm{eff}}\left(z,\theta \right)=\frac{A_{t}}{z_{\max}}z+\pi \sin^{2}\theta \cos\theta \;z\left(z_{\max}-z\right).$$

Under fixed supply pressure and temperature, and within this preliminary continuum model, the static thrust-displacement relation is approximated by

(9) $$F_{\mathrm{th}}\left(z,\theta \right)=F_{d}\frac{A_{\mathrm{eff}}\left(z,\theta \right)}{A_{t}},$$

where $F_{\mathrm{th}}$ is the preliminary theoretical thrust corresponding to displacement *z* and half-angle θ. Equation (8) directly identifies the geometric source of nonlinearity: the second-order term is controlled by $\sin^{2}\theta \cos\theta$. A smaller needle half-angle improves linearity but increases the required inlet-orifice diameter in Equation (7), whereas a larger half-angle leads to a more compact structure at the cost of stronger curvature in the displacement-thrust curve.

**Figure 2:**
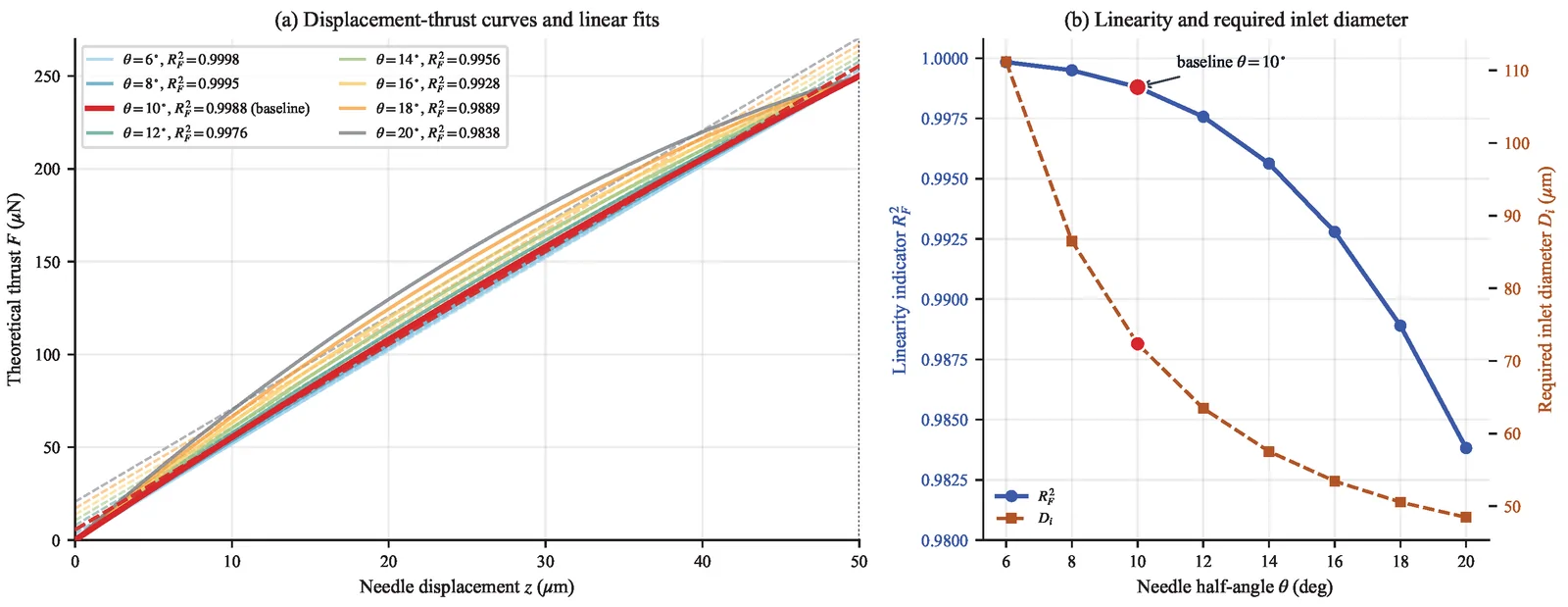
Theoretical displacement-thrust curves for different needle half-angles and their linear fits. The scan uses θ=6–$20^{{}^{\circ}}$ in $2^{{}^{\circ}}$ increments, $p_{c}=1\;\mathrm{bar}$, $T_{c}=298.15\;K$, $z_{\max}=50\;\mu m$, and ϵ=25; the selected theoretical baseline is $\theta =10^{{}^{\circ}}$.

For each candidate needle half-angle, the linearity is evaluated by fitting $F_{\mathrm{fit}}\left(z\right)=a_{\theta }z+b_{\theta }$ and computing

(10) $$R_{F}^{2}=1-\frac{\sum_{i=1}^{N}{\left[F_{\mathrm{th}}\left(z_{i},\theta \right)-F_{\mathrm{fit}}\left(z_{i}\right)\right]}^{2}}{\sum_{i=1}^{N}{\left[F_{\mathrm{th}}\left(z_{i},\theta \right)-{\bar{F}}_{\mathrm{th}}\right]}^{2}},$$

where *N* is the number of sampled displacement points, $z_{i}$ is the *i*th sampled displacement, $F_{\mathrm{fit}}$ is the fitted linear thrust, and ${\bar{F}}_{\mathrm{th}}$ is the mean theoretical thrust over the sampled points. Figure 2 shows the theoretical displacement-thrust curves for θ=6–$20^{{}^{\circ}}$ in $2^{{}^{\circ}}$ increments, together with the corresponding linear fits and $R_{F}^{2}$ values. The results confirm the expected trade-off: $R_{F}^{2}$ decreases from 0.9998 at $6^{{}^{\circ}}$ to 0.9838 at $20^{{}^{\circ}}$, while the required $D_{i}$ decreases from approximately 111.20 μm to 48.46 μm. Considering linearity, manufacturing feasibility, and structural compactness, $\theta =10^{{}^{\circ}}$ is selected as the theoretical baseline for the following analysis. This baseline retains a high linearity of $R_{F}^{2}=0.9988$, reduces the required inlet diameter to $D_{i}=72.36\;\mu m$, and gives a local theoretical sensitivity range of 4.33–5.67 μN/μm over the 0–50 μm stroke.

Based on the selected $\theta =10^{{}^{\circ}}$ theoretical baseline, the fixed geometric parameters used in the subsequent displacement-thrust analysis are summarized in Table 3.

## 3. DSMC Numerical Simulation and Optimization

### 3.1. DSMC Numerical Simulation Analysis

The theoretical calculation in Section 2.1.2 provides an initial estimate of the nozzle scale and the static displacement–thrust relation, but it remains a one-dimensional isentropic model. This approximation is insufficient for the present integrated microthruster head, in which the actual flow path includes a finite cone-needle/orifice throttling gap, a short converging section, a micron-scale throat, strong gas–wall interactions, and pronounced rarefaction gradients from the throttling region to the plume. Therefore, the complete flow is governed not only by compressibility but also by the detailed local geometry, and the pressure and velocity fields cannot be represented accurately by a one-dimensional model based on a nominal upstream pressure.

**Figure 3:**
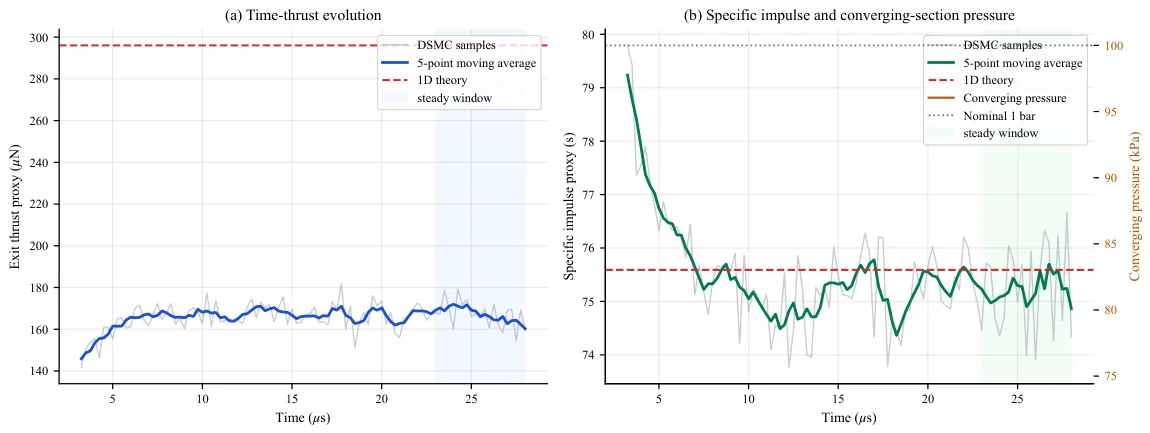
Time histories of the thrust, specific impulse, and converging-section pressure for the full-opening DSMC reference case with $R_{t}=23.537\;\mu m$, $\theta =10^{{}^{\circ}}$, and z=50 μm. The dashed or dotted line in each panel is the corresponding one-dimensional theoretical or nominal pressure reference.

To assess the theoretical baseline in the complete throttle–nozzle geometry, a DSMC reference simulation was carried out for the full-opening case with $R_{t}=23.537\;\mu m$, $\theta =10^{{}^{\circ}}$, and z=50 μm under a nominal 1 bar inlet condition. As shown in Figure 3, the solution reaches a stable plateau after the initial transient. Over the steady interval of 23–28 μs, the exit-plane mass flow rate and thrust are $\left(2.267\pm 0.085\right)\times 10^{-7}\;\mathrm{kg}/s$ and 167.29±6.45 μN, respectively, compared with the one-dimensional theoretical estimates of $3.994\times 10^{-7}\;\mathrm{kg}/s$ and 296.05 μN. The specific impulse remains close to the theoretical value, 75.24±0.86 s versus 75.59 s, indicating that the expansion section still converts the transmitted mass flow into axial momentum efficiently. Quantitatively, the DSMC mass flow and thrust correspond to approximately 56.8% and 56.5% of the one-dimensional estimates, respectively, whereas the specific-impulse difference is only about 0.46%.

The reduction in mass flow and thrust mainly arises because the effective pressure entering the converging section is only about 76.34±0.28 kPa, or 76.3% of the nominal upstream pressure. Thus, the one-dimensional model overestimates the available mass flow by neglecting pressure drop, wall-collision loss, entrance contraction loss, and local nonequilibrium effects in the integrated micro-scale geometry. This result indicates that the preliminary one-dimensional throat size does not provide sufficient flow capacity in the integrated head geometry; therefore, the subsequent DSMC-based optimization uses a larger $R_{t}$ to recover the required mass flow and satisfy the 200 μN upper-thrust requirement.

### 3.2. DSMC-Based Parameter Optimization

The complete-geometry DSMC baseline shows that the preliminary one-dimensional sizing overestimates the available flow capacity when the throttle and nozzle are treated as an integrated device. Therefore, the DSMC-based optimization was performed in two stages. First, the coupled parameter pair $\left(R_{t},\theta \right)$ was scanned under the maximum-opening condition to retain coarse candidates with large-opening thrust in the 200–220 μN target window. Second, representative candidates were evaluated at multiple throttling openings to compare their displacement–thrust curves and fitted linearity, from which the final design parameters were selected.

#### 3.2.1. Step 1: Maximum-Opening Screening in the Throat-Radius/Needle-Angle Space

**Figure 4:**
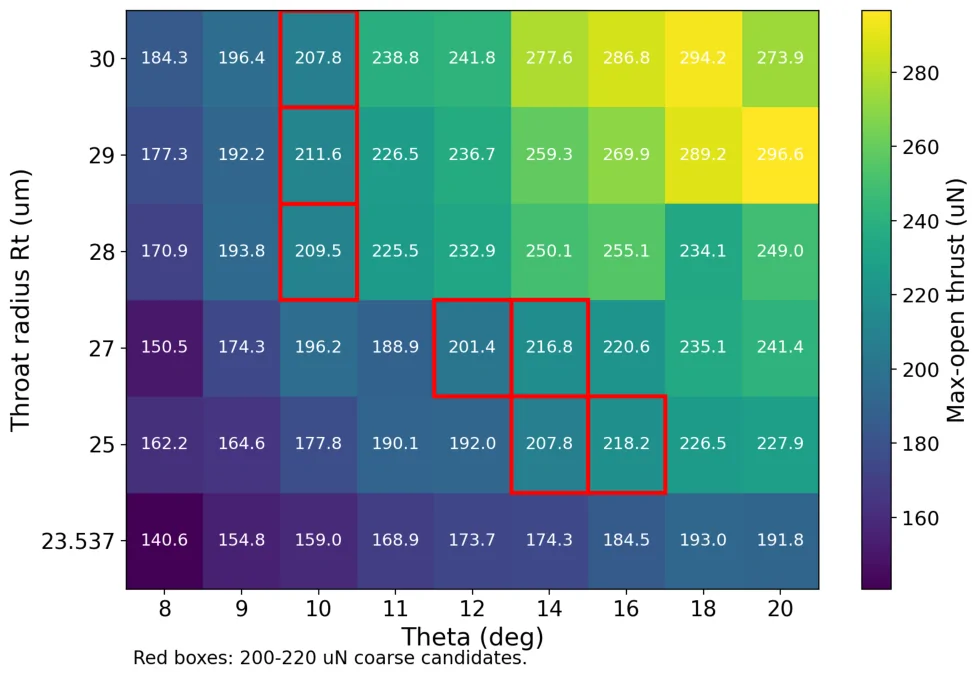
DSMC-based $R_{t}$–θ candidate screening map under a representative large-opening condition. Red boxes indicate coarse candidate combinations whose maximum-opening thrust falls within the 200–220 μN window.

In the first stage, full-open or large-opening DSMC cases were used to screen throat radii and needle half-angles that could satisfy the required upper-thrust range after complete-geometry correction. Figure 4 summarizes the updated $R_{t}$–θ screening map obtained from the recomputed DSMC results. The red boxes mark the coarse candidate combinations whose maximum-opening thrust falls within the 200–220 μN window. The feasible candidates exhibit a coupled geometric trade-off: increasing $R_{t}$ generally enhances the nozzle-side flow capacity, while increasing θ changes the throttling-area sensitivity and can compensate for a smaller throat radius.

#### 3.2.2. Step 2: Multi-Opening Comparison and Linearity Optimization

In the second stage, representative candidates from the first-stage feasible set were re-simulated at multiple throttling displacements to obtain their full displacement–thrust relations. The resulting static maps were compared using the fitted linearity $R_{F}^{2}$, with the same definition as in Equation (10). Here, $R_{F}^{2}\geq 0.95$ is used as an acceptable screening threshold and $R_{F}^{2}\geq 0.98$ as a preferred threshold for candidate comparison; the final flow and thrust characteristics are still verified through calibration after fabrication.

**Figure 5:**
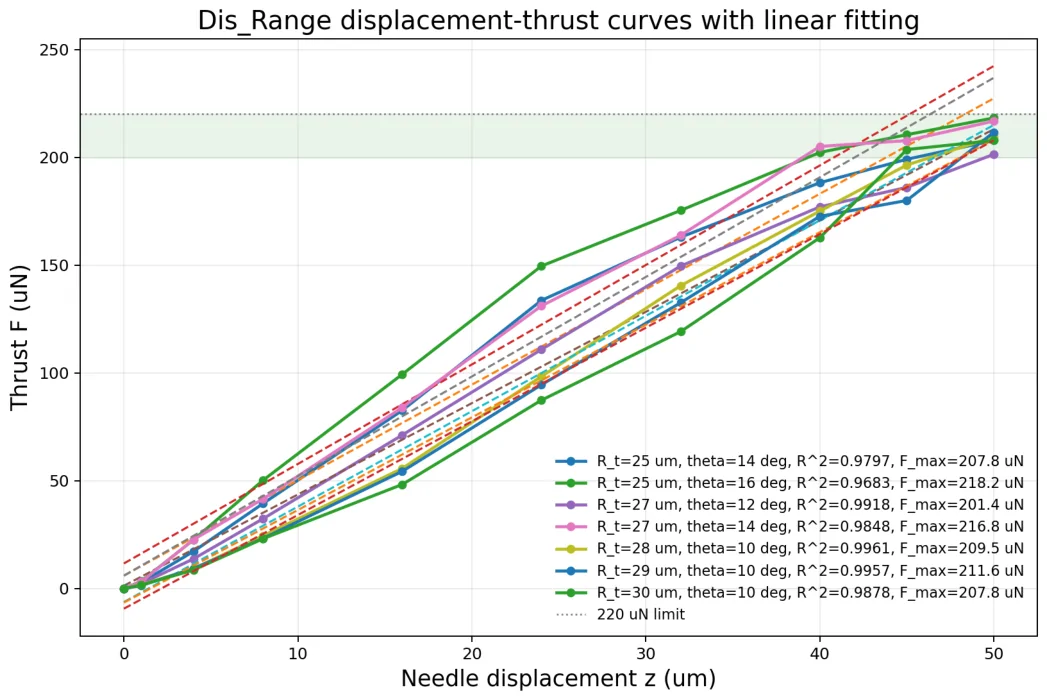
Predicted displacement-thrust curves and linear fits for representative DSMC candidates retained after Step 1. The $R_{t}=28\;\mu m$, $\theta =10^{{}^{\circ}}$ and $R_{t}=29\;\mu m$, $\theta =10^{{}^{\circ}}$ cases show comparable fitted linearity, while the latter is selected as the updated main scheme because of its slightly higher maximum thrust.

The candidate comparison summarized in Table 4 and Figure 5 confirms that the final design must balance upper-thrust reserve and displacement–thrust linearity rather than maximize either metric alone. Among the compared candidates, the $R_{t}=28\;\mu m$, $\theta =10^{{}^{\circ}}$ and $R_{t}=29\;\mu m$, $\theta =10^{{}^{\circ}}$ cases yield very similar fitted linearity, with $R^{2}=0.9961$ and $R^{2}=0.9957$, respectively. Because the $R_{t}=29\;\mu m$, $\theta =10^{{}^{\circ}}$ case provides a slightly higher maximum thrust of 211.60 μN compared with 209.51 μN for the $R_{t}=28\;\mu m$, $\theta =10^{{}^{\circ}}$ case, this configuration was selected as the main DSMC baseline for the subsequent design update.

### 3.3. Predicted Flow-Displacement and Thrust-Displacement Characteristics

The final DSMC sweep was used to characterize the predicted flow–displacement and thrust–displacement behavior of the selected $R_{t}=29\;\mu m$, $\theta =10^{{}^{\circ}}$ design over the working range. Representative displacements of z=1, 4, 8, 16, 32, and 50 μm were selected to span the transition from near-closed throttling to the maximum-opening state. These cases define the quantities subsequently verified in the experiments, including the formation accuracy of the key micron-scale flow features, the command-to-flow calibration, and the flow-to-thrust conversion of the integrated head.

Table 5 summarizes the DSMC-predicted operating points of the selected design using the nozzle-exit flow as the representative flow variable. The results show a monotonic increase in flow rate and thrust with displacement, while the chamber pressure remains close to the nominal 1 bar inlet boundary condition.

**Figure 6:**
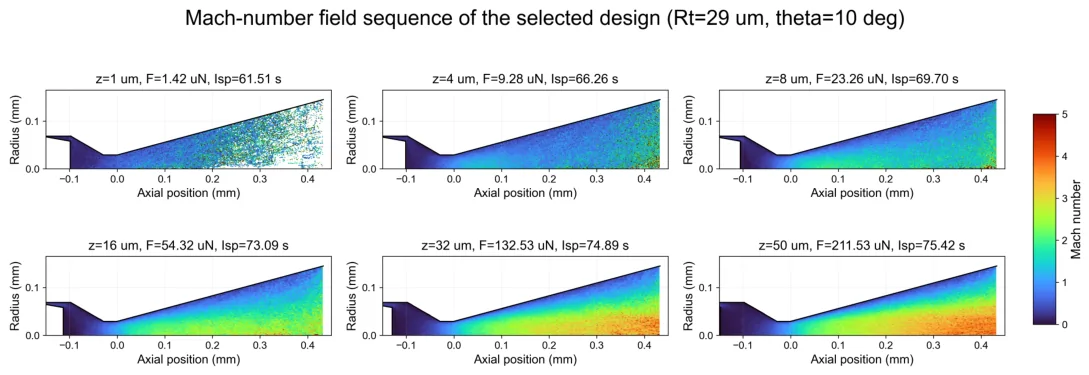
DSMC-predicted Mach-number field sequence for the selected $R_{t}=29\;\mu m$, $\theta =10^{{}^{\circ}}$ head geometry at representative throttling displacements, linking opening displacement, predicted thrust, and nozzle expansion behavior.

**Figure 7:**
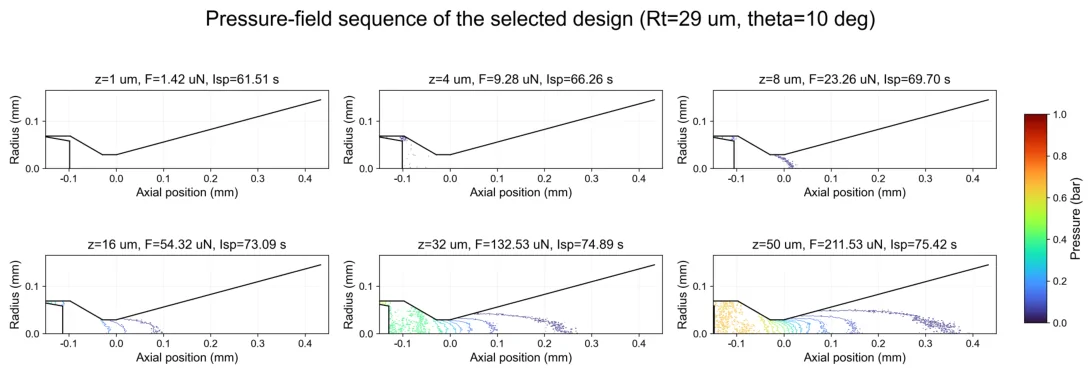
DSMC-predicted pressure-field sequence for the selected $R_{t}=29\;\mu m$, $\theta =10^{{}^{\circ}}$ head geometry at representative throttling displacements, showing how the dominant pressure drop evolves from the throttling interface to the nozzle expansion section.

Figure 6 and Figure 7 jointly illustrate how the flow field develops as the throttling displacement increases. At small openings, most of the pressure drop is concentrated across the cone-needle/orifice restriction, leaving only a weak high-Mach core in the expansion section. As the displacement increases, the throttling loss weakens, the pressure recovery upstream of the throat improves, and a more continuous pressure-release path forms through the converging and expansion sections. Correspondingly, the high-Mach region extends through most of the nozzle passage at large openings, indicating stronger axial acceleration and more efficient conversion of the available pressure energy into jet momentum.

**Figure 8:**
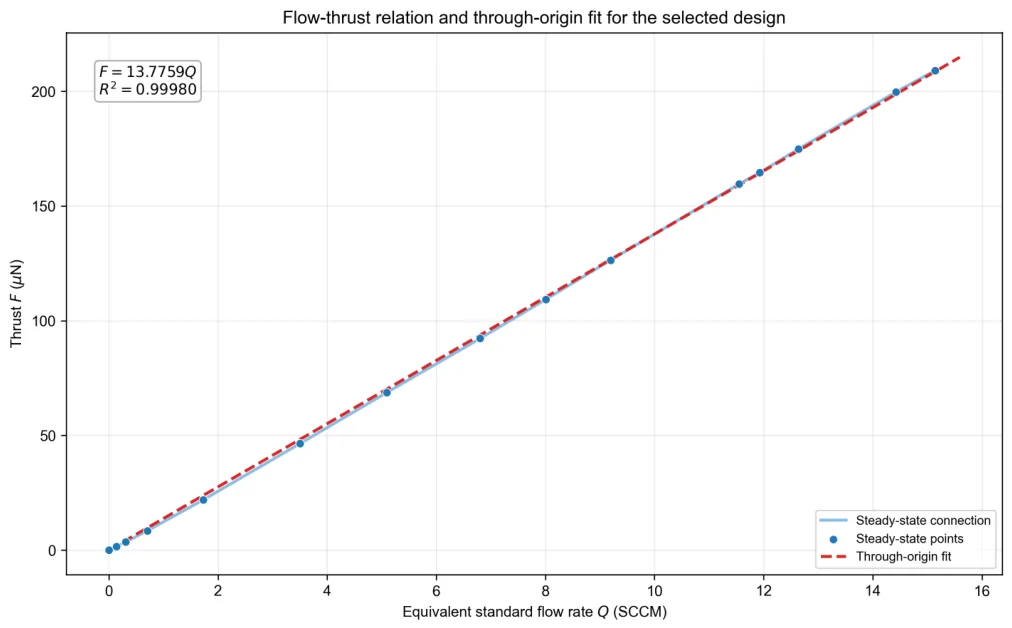
Predicted flow–thrust relation for the selected $R_{t}=29\;\mu m$, $\theta =10^{{}^{\circ}}$ configuration, together with a through-origin linear fit for later experimental comparison.

Figure 8 shows the DSMC-predicted relation between the equivalent standard flow rate *Q* and thrust for the selected design. Based on the simulation dataset used for the subsequent experimental comparison, the through-origin fitted relation is F=13.7759Q with $R^{2}=0.99980$, where *Q* is in SCCM and *F* is in μN. This curve provides the simulation-based flow–thrust reference for the later experimental validation.

## 4. Fabrication and Characterization of Key Components

### 4.1. System-Level Arrangement of the Micropropulsion Device

**Figure 9:**
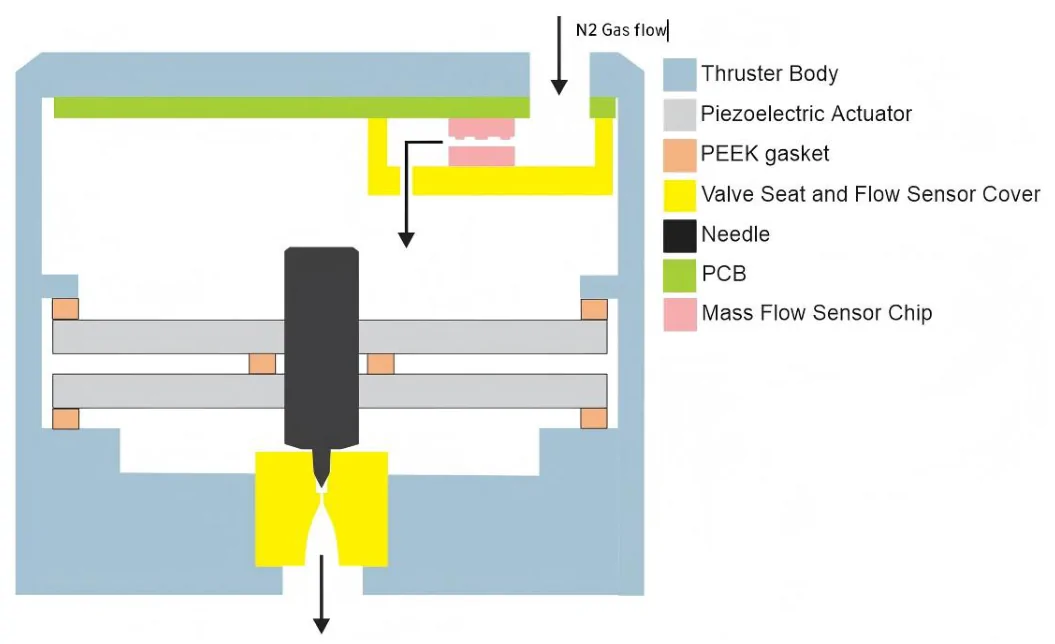
System-level arrangement of the piezoelectrically controlled cold-gas microthruster head. The module integrates the thruster body, piezoelectric actuator, polyether ether ketone (PEEK) gaskets, valve seat and flow-sensor cover, cone needle, printed circuit board (PCB), and thermal mass-flow sensor chip into a compact gas-flow and actuation path.

The microthruster is designed as a compact head module that integrates the gas-supply path, piezoelectric actuation path, cone-needle throttling interface, thermal mass-flow sensing path, and downstream nozzle. As shown in Figure 9, the thruster body provides the mechanical reference and gas-tight housing, while the piezoelectric actuator is arranged along the central axis to drive the needle axially. The valve seat and flow-sensor cover define the local gas passage, and the cone-needle/seat interface forms the controllable throttling section directly upstream of the nozzle.

Within this integrated-head layout, the gas path and actuation path are directly coupled. The incoming gas first passes through the upper internal channel and the thermal mass-flow sensing region, where the integrated sensor provides the flow-feedback signal used for indirect thrust regulation. The gas is then routed to the valve-seat region, where the axial position of the cone needle determines the effective throttling area. After passing through this controllable restriction, the flow enters the downstream micro-nozzle and is converted into axial momentum at the outlet. In this way, the module establishes a compact command–flow–thrust chain inside the thruster head.

The axial motion of the cone needle is provided by a CMBR05 piezoelectric actuator manufactured by CoreMorrow. The actuator is driven over a voltage range of ±100 V, corresponding to a nominal free displacement range of approximately ±70 μm. In the assembled thruster head, the effective needle motion can differ from the free-stroke actuator specification because of preload, assembly constraints, actuator hysteresis, and mechanical coupling between the actuator and the cone needle. Therefore, the control strategy and performance evaluation in this work do not rely solely on an open-loop voltage–displacement relation. Instead, the effective actuation behavior is calibrated at the assembled-head level through the measured relationships among the lead zirconate titanate piezoelectric actuator (PZT) drive command, mass-flow output, and thrust output. During operation, the integrated mass-flow sensor provides feedback to the controller, and the PZT driving voltage is adjusted by the PID flow-control algorithm.

### 4.2. Design and Fabrication of the Integrated Throttle-Nozzle Valve Seat

**Figure 10:**
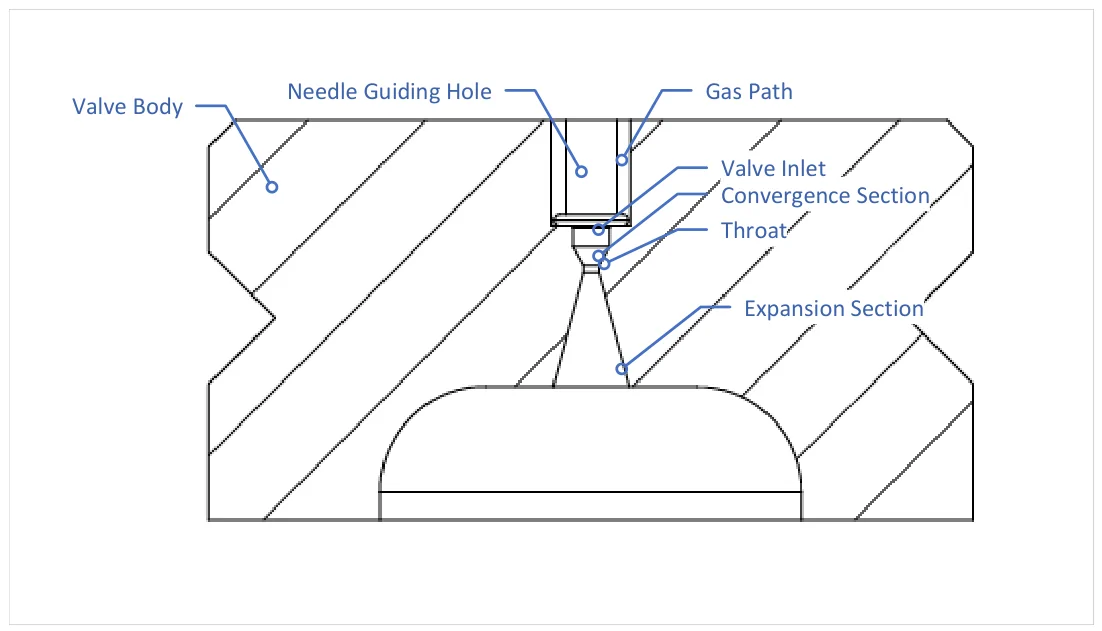
Detailed cross-sectional design of the integrated throttle-nozzle structure, showing the needle guide bore, gas channel, throttling section, converging section, throat, and expansion section.

The microthruster head uses an integrated throttle–nozzle valve-seat structure. As shown in Figure 10, the gas path delivers the propellant gas to the valve-inlet region, the needle guide hole constrains the cone needle along the nozzle axis, and the downstream passage consists of the converging section, micron-scale throat, and expansion section. This compact coaxial layout provides the structural basis for coupling displacement-controlled throttling with downstream nozzle acceleration.

Because this structure contains micron-scale internal passages and requires coaxial alignment of the needle guide hole, throttling interface, throat, and expansion section, conventional machining or multi-piece assembly is difficult to implement. Therefore, the integrated valve seat was fabricated using projection micro-stereolithography (PμSL), a high-resolution vat-photopolymerization 3D micro-printing process. The PμSL process provides an optical printing accuracy of about 2 μm, satisfying the manufacturing requirements of the integrated micro-scale throttle–nozzle geometry. Printing the valve seat as a single body forms the guide, inlet, throat, and expansion features in one component, thereby reducing secondary alignment error and avoiding an additional sealing interface between the throttle and nozzle.

**Figure 11:**
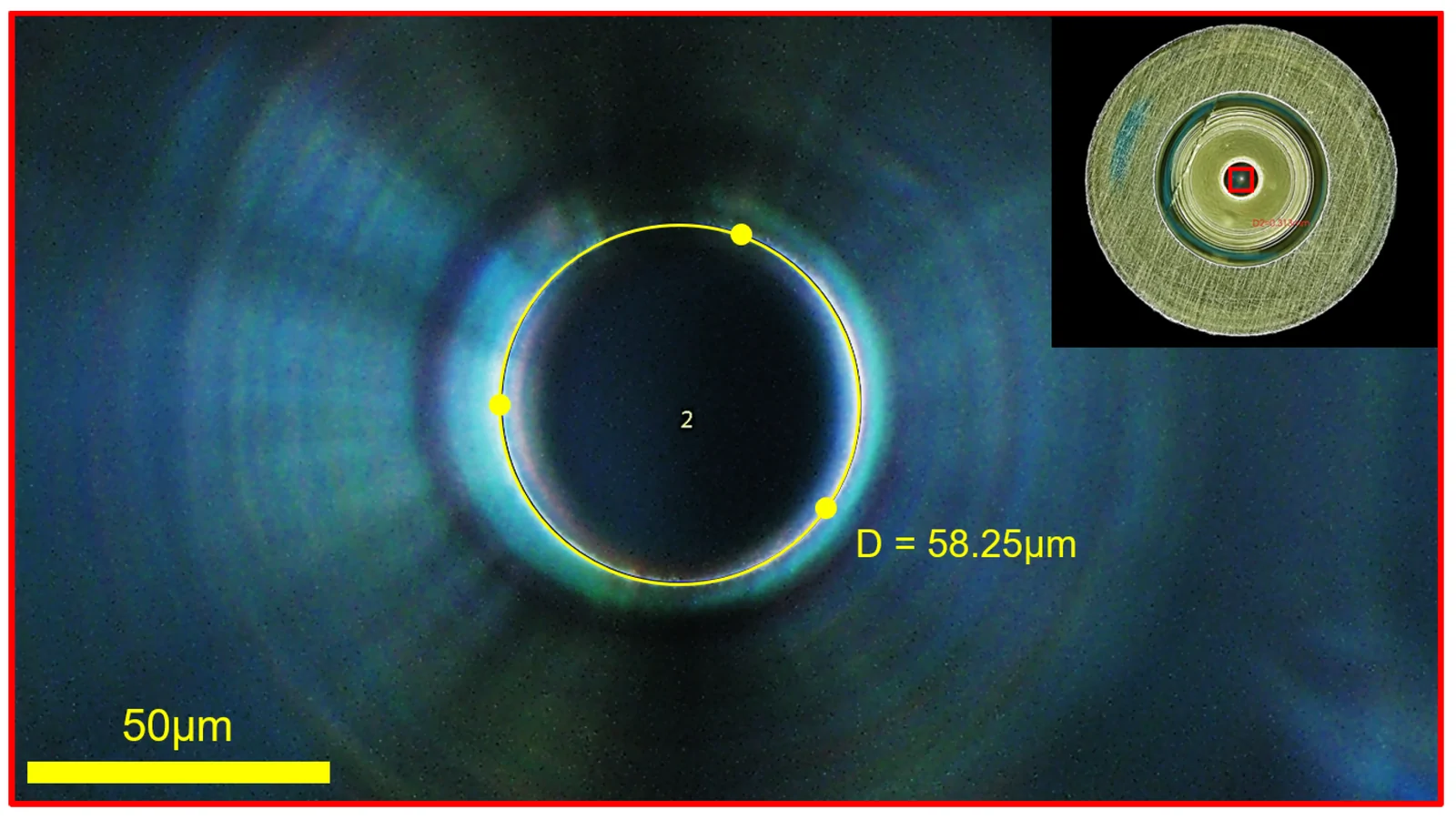
Microscope image of the throat-scale micro-hole in the printed integrated valve seat. The measured central opening diameter is 58.25 μm, with a 50 μm scale bar shown in the image.

After PμSL printing and cleaning, the throat-scale micro-hole of the integrated valve seat was optically inspected before assembly. Figure 11 shows a representative microscope image, where the measured central opening diameter is 58.25 μm. This value is close to the selected design throat diameter of approximately 58 μm ($R_{t}=29\;\mu m$), indicating that the key micron-scale flow feature was successfully formed.

### 4.3. Integrated Mass-Flow Measurement Path

The thruster head also includes an internal thermal mass-flow measurement path. The sensor is integrated into the gas path to measure the flow supplied to the downstream throttle–nozzle structure, and its calibrated output is acquired by the controller for subsequent flow-feedback control.

To improve dynamic response, the calibrated mass-flow sensor is placed close to the throttling valve so that the measured flow signal more directly reflects the gas supplied to the nozzle. The signal-conditioning circuit is also arranged near the sensor, reducing interference during weak bridge-signal transmission and improving flow-signal acquisition accuracy.

**Figure 12:**
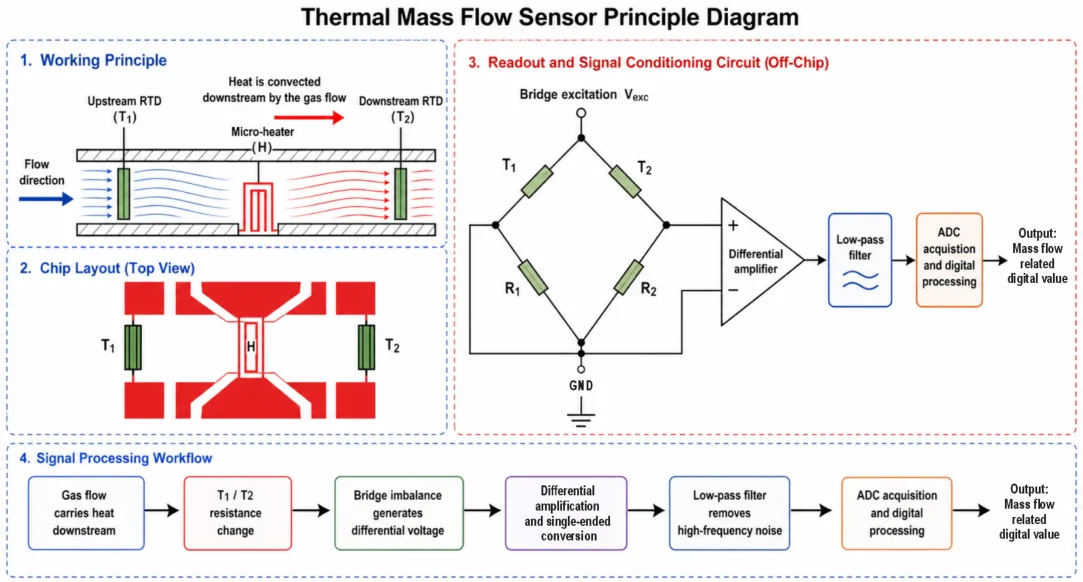
Thermal mass-flow sensing principle and readout chain used in the integrated thruster head. The sensor combines a micro-heater, upstream and downstream temperature-sensitive resistors, bridge readout, differential amplification, filtering, and digital acquisition.

**Figure 13:**
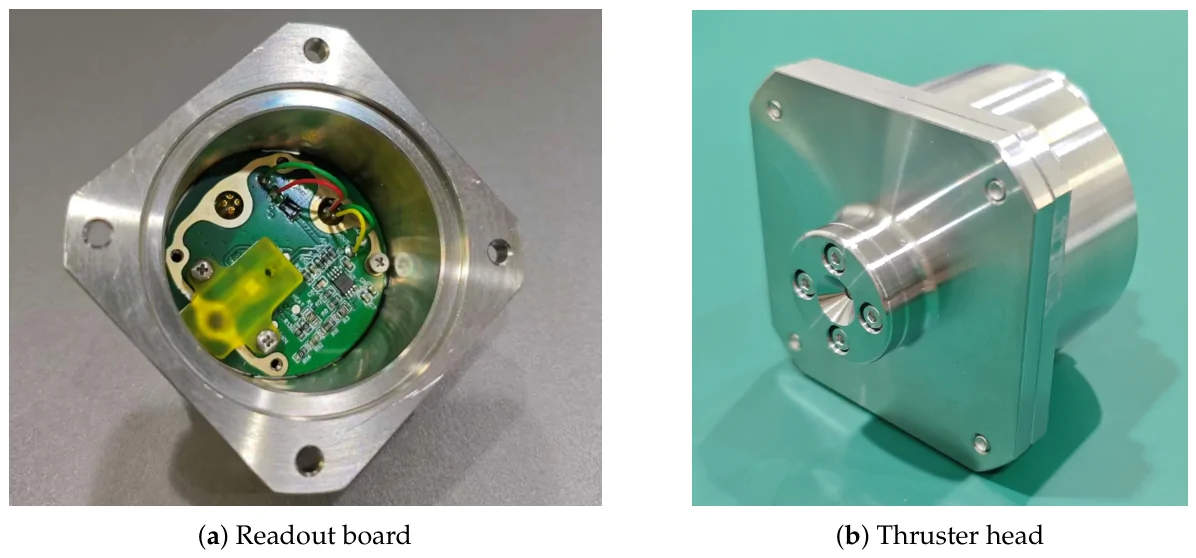
Hardware photographs of the integrated mass-flow measurement path and assembled thruster head. The readout board performs local bridge-signal amplification and conditioning before acquisition by the controller, while the assembled head integrates the gas path, piezoelectric actuation path, valve seat, and downstream nozzle into one compact module.

The thermal flow sensor uses a MEMS-based thermal flow-sensing principle. A micro-heater and two thermistors are arranged on opposite sides of the heater, and their resistance changes are detected via a Wheatstone bridge. The bridge differential voltage is mapped to the temperature difference between the two thermistors, amplified by a high-precision operational-amplifier circuit, and then sent to the controller as the flow-feedback signal. This sensing and readout chain is illustrated in Figure 12, and the corresponding near-sensor readout board is shown in Figure 13a.

The readout board is installed close to the mass-flow sensing chip inside the thruster-head housing, providing local bridge-signal amplification and conditioning before acquisition by the controller.

### 4.4. Integrated Head Assembly Summary

The fabricated and assembled cold-gas microthruster head is shown in Figure 13b. The final module integrates the PμSL-printed throttle-nozzle valve seat, coaxial piezoelectric needle-actuation path, MEMS thermal mass-flow sensing path, and near-sensor signal-conditioning electronics within a compact integrated-head package. This integrated assembly provides the hardware basis for the subsequent flow calibration and thrust-output validation.

At the control level, the integrated mass-flow meter provides the direct feedback signal for regulating the thruster head. After calibration, the sensor output voltage is converted into the calibrated flow rate and compared with the target flow corresponding to the required thrust level.

The controller then adjusts the voltage of the piezoelectric actuator to change the needle displacement and the effective throttling gap of the integrated throttle-nozzle valve seat. In this way, the control loop does not rely on direct real-time thrust measurement; instead, it uses flow regulation and the calibrated actuator–flow–thrust relationship to achieve indirect thrust control.

## 5. Mass-Flow Calibration and Thrust Test

### 5.1. Mass-Flow Calibration

#### 5.1.1. Mass-Flow Measurement and Calibration Method

The mass-flow calibration test was performed to relate both the analog voltage output of the integrated MEMS thermal mass-flow meter and the piezoelectric drive command to the delivered flow rate in the assembled throttling configuration. The calibration setup included a nitrogen supply, pressure-regulation module, assembled thruster head, piezoelectric drive circuit, temperature measurement, internal mass-flow meter, and external ground-reference flow meter. During ground calibration, the reference flow meter provided an independent measurement of the delivered flow rate and was used to calibrate the voltage output of the internal mass-flow measurement chain.

Two complementary calibration paths were used to improve the traceability and reliability of the mass-flow calibration. The ground-reference path directly relates the internal flow-meter voltage to the delivered flow rate measured by an external reference instrument, providing a straightforward laboratory calibration. The pressure-decay path estimates flow rate from the pressure decay of a calibrated gas volume using the pressure and temperature sensors already integrated in the thruster system. After ground calibration of the effective volume and pressure-sensor channel, this path can also support later in-orbit recalibration without relying on an external flow meter. The agreement between the two paths is used as a cross-check for the integrated mass-flow measurement chain.

For the ground-reference calibration path, the inlet-pressure boundary was first stabilized, and the piezoelectric drive voltage was then varied step by step to obtain a series of steady flow points. At each operating point, the analog voltage output of the internal mass-flow meter and the reading of the ground-reference flow meter were recorded after the flow reached a steady state, while pressure and temperature were monitored as supporting quantities. Zero-flow measurements were used to evaluate the offset and drift of the internal sensing channel.

For the present study, the pressure-decay path was adopted as the primary calibration route, whereas the ground-reference flow meter was used for external verification. Accordingly, the analog voltage output of the internal mass-flow sensor, $V_{\mathrm{mfs}}$, was converted to the unified flow variable *Q* through a calibration function determined from the pressure-decay data,

(11) $$Q=a_{0}+a_{1}V_{\mathrm{mfs}}+a_{2}V_{\mathrm{mfs}}^{2}+a_{3}V_{\mathrm{mfs}}^{3},$$

where $a_{i}$ are fitted calibration coefficients. The ground-reference measurements were then compared with this fitted relation to evaluate calibration consistency.

**Figure 14:**
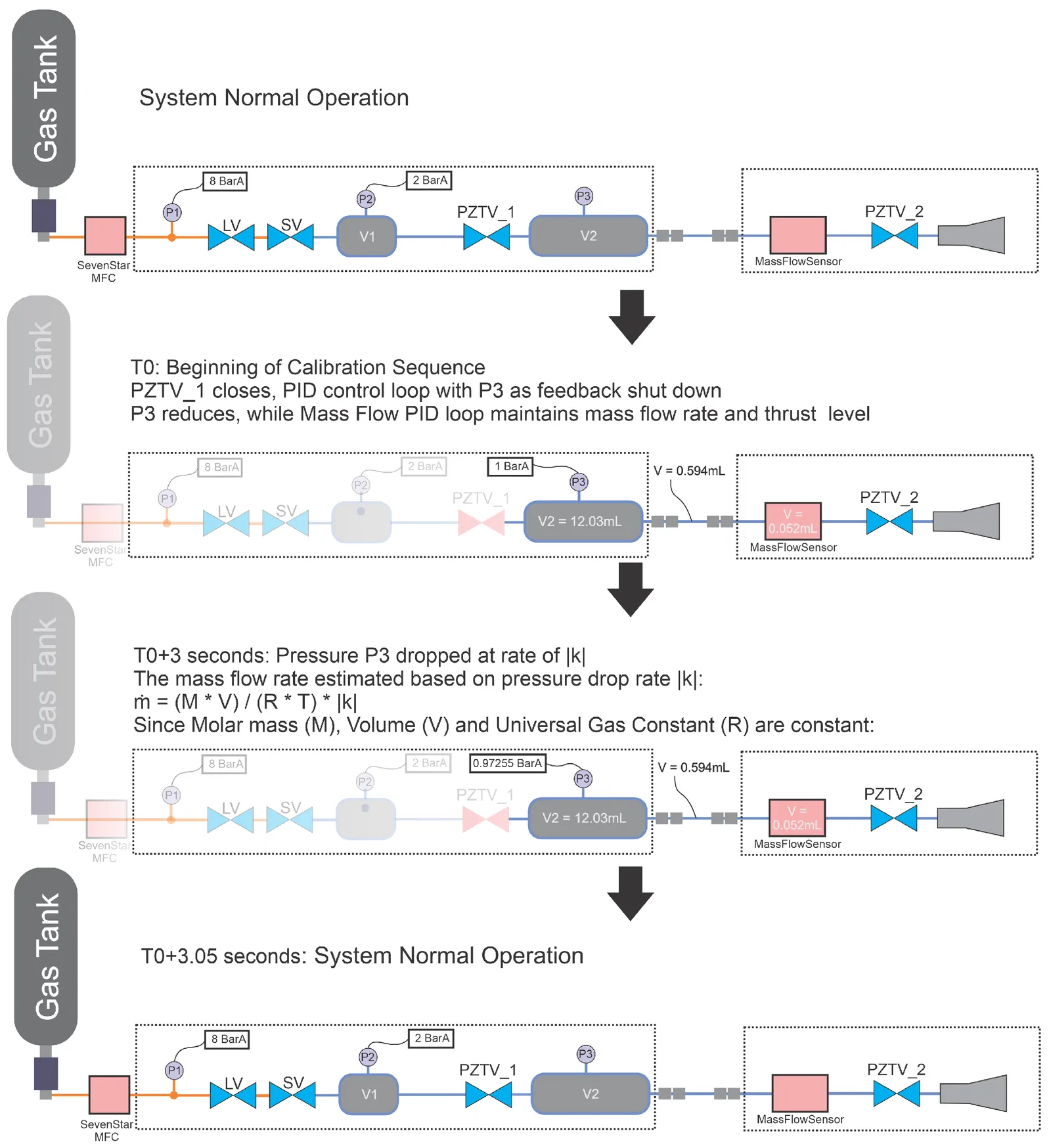
Dual-path mass-flow calibration concept for the piezoelectrically controlled thruster head. The pressure-decay path uses the pressure and temperature sensors integrated in the thruster system, whereas the ground-reference path uses an external flow meter to provide an independent laboratory reference.

For the pressure-decay calibration path, the flow rate was estimated from the pressure decay of a calibrated gas volume, as summarized in Figure 14. During this test, the throttle operating command was held constant, the upstream regulation branch was temporarily isolated, and the pressure decay in the selected internal volume was recorded over a short interval. The pressure-decay rate was first converted to mass flow using the gas properties, gas temperature, and calibrated effective volume. The corresponding estimate is

(12) $$\dot{m}=\frac{MV_{\mathrm{cal}}}{RT}\left|\frac{dP}{dt}\right|,$$

where *M* is the molar mass of the working gas, $V_{\mathrm{cal}}$ is the calibrated effective gas volume associated with the monitored decay segment, *R* is the universal gas constant, *T* is the gas temperature, and dP/dt is the measured absolute-pressure decay rate. For consistency with the subsequent flow–thrust comparison, the estimated mass-flow values were further converted to the equivalent standard volumetric flow rate *Q*, expressed in standard cubic centimeters per minute (SCCM), which is used as the unified flow variable in the following calibration and thrust-analysis sections. Because this path relies on the pressure and temperature sensors integrated in the thruster system, it provides an independent check of the internal mass-flow measurement chain and can support later in-orbit recalibration after ground characterization.

#### 5.1.2. Mass-Flow Calibration Results

**Figure 15:**
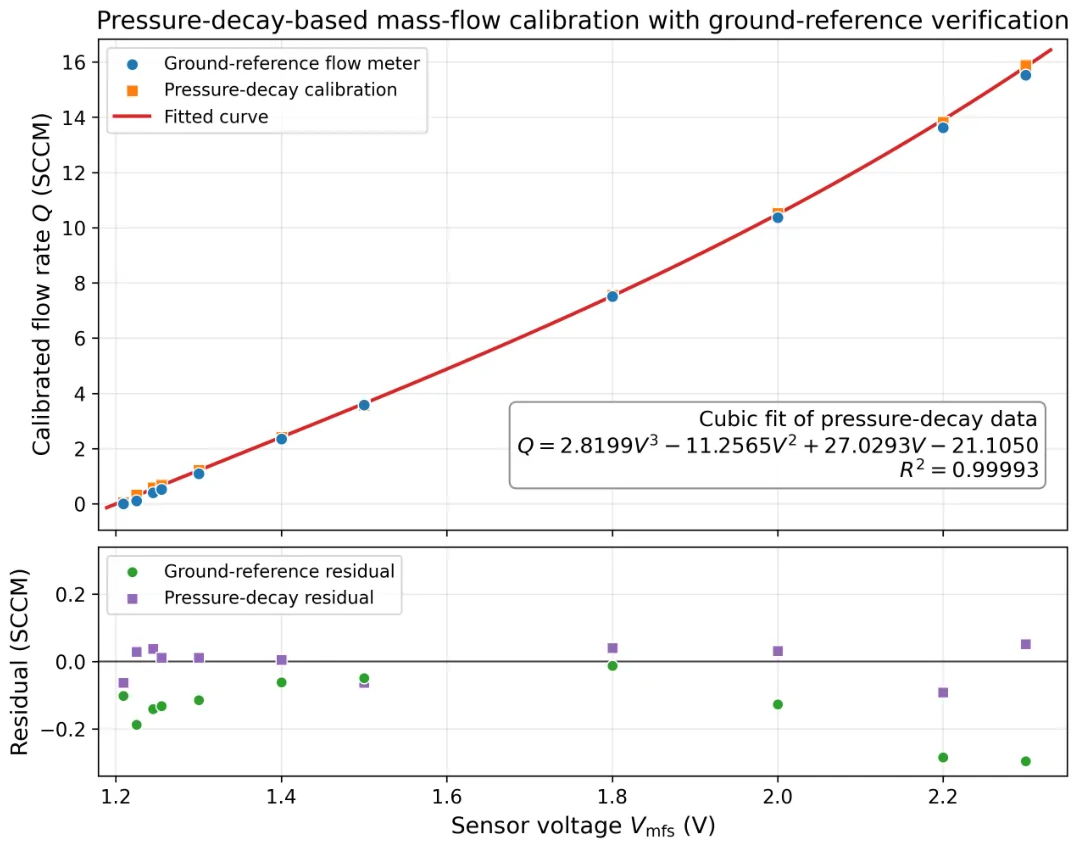
Pressure-decay-based mass-flow calibration of the integrated MEMS sensor, with the ground-reference flow meter used for external verification. The upper panel shows the cubic calibration curve fitted from the pressure-decay data together with the ground-reference measurements, and the lower panel compares the residuals of the two calibration paths relative to the fitted pressure-decay-based curve.

The mass-flow calibration results are summarized in Figure 15. The pressure-decay method was adopted as the primary calibration path to establish the relationship between the internal sensor voltage and the calibrated flow rate, while the ground-reference flow meter was used as an independent reference for consistency verification. Based on the pressure-decay data, the calibration curve was fitted by a cubic polynomial,

(13) $$Q=2.8199V^{3}-11.2565V^{2}+27.0293V-21.1050,$$

with a coefficient of determination of $R^{2}=0.99993$, indicating that the pressure-decay-based calibration provides a stable mapping over the tested flow range.

The residual distributions of the two paths are also compared in Figure 15. The pressure-decay calibration points remain close to the fitted curve, with calibration residuals ranging from −0.091 to +0.051 SCCM, indicating internal consistency of the adopted calibration path. The corresponding root-mean-square residual is approximately 0.047 SCCM, which is below 0.3% of the maximum calibrated flow in the present dataset. This residual level provides an estimate of the uncertainty of the calibrated flow variable used in the subsequent thrust and specific-impulse evaluations. The ground-reference flow-meter data follow the same overall trend and provide an external cross-check of the calibration result. Although noticeable deviations appear toward the ends of the tested flow range, the agreement is sufficient for using the pressure-decay curve as the unified flow reference in the present tests.

### 5.2. Flow-Feedback Control Algorithm

During operation, the controller regulates thrust indirectly through flow feedback. The target thrust is first converted into a target flow rate using the calibrated flow–thrust relation. The integrated thermal mass-flow sensor measures the delivered flow rate, and the flow error is calculated as the difference between the target and measured flow rates. A PID controller then updates the driving voltage applied to the CMBR05 piezoelectric actuator. The actuator changes the cone-needle displacement and the effective throttling gap, thereby adjusting the delivered flow rate. In this way, the thrust command is executed through closed-loop flow regulation rather than through direct real-time thrust measurement.

In discrete form, the PZT driving-voltage command can be written as

(14) $$u\left[k\right]=u_{0}+K_{p}e\left[k\right]+K_{i}\sum_{j=0}^{k}e\left[j\right]\Delta t+K_{d}\frac{e[k]-e[k-1]}{\Delta t},$$

where u[k] is the PZT driving voltage at the *k*th sampling instant, $u_{0}$ is the bias voltage, $e\left[k\right]=Q_{\mathrm{ref}}\left[k\right]-Q\left[k\right]$ is the calibrated flow-rate error, $Q_{\mathrm{ref}}$ is the target flow rate corresponding to the required thrust command, *Q* is the calibrated flow rate measured by the integrated mass-flow sensor, $K_{p}$, $K_{i}$, and $K_{d}$ are the proportional, integral, and derivative gains, respectively, and Δt is the sampling interval. The output voltage is limited to the actuator driving range of ±100 V.

### 5.3. Thrust Test

#### 5.3.1. Thrust Test Method and Facility

**Figure 16:**
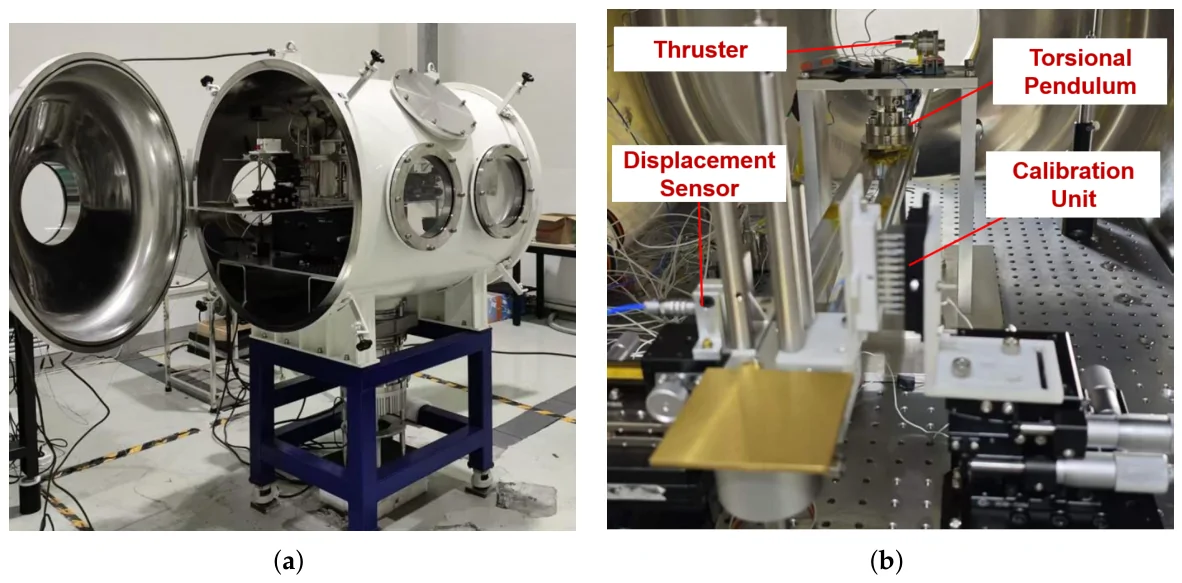
Thrust-test facility for the fabricated cold-gas microthruster head: (**a**) overall vacuum thrust-test system; (**b**) torsional pendulum micro-thrust stand showing the thruster, displacement sensor, pendulum structure, and calibration unit.

The thrust performance of the fabricated thruster head was tested in vacuum using a torsional-pendulum micro-thrust stand, as shown in Figure 16. During the test, the chamber pressure was maintained at approximately $10^{-2}\;\mathrm{Pa}$, so the back-pressure effect on nozzle expansion could be neglected. The thruster was mounted on the pendulum inside the vacuum chamber, and the output thrust produced a pendulum displacement. The displacement was measured by a non-contact sensor and then converted into thrust through prior calibration of the pendulum system.

Figure 16a shows the overall vacuum thrust-test facility, including the vacuum chamber, thrust stand, thruster mounting interface, and supporting gas and electrical feedthroughs. Figure 16b shows the torsional-pendulum assembly in detail. The thruster was installed on the pendulum arm, the displacement sensor was used to monitor the pendulum response, and the calibration unit was used to establish the displacement-thrust relationship for subsequent thrust measurement.

Before thrust testing, the thrust stand was calibrated using a known force input, and the measured displacement response was converted to thrust by

(15) $$F\left(t\right)=K_{\mathrm{cal}}\Delta y\left(t\right),$$

where $K_{\mathrm{cal}}$ is the thrust-stand calibration coefficient and Δy(t) is the measured stand displacement.

#### 5.3.2. Thrust Test Results and Analysis

Using the unified calibrated flow variable established above, the thrust-test results are analyzed in terms of four metrics: flow–thrust correspondence, specific impulse, thrust resolution, and thrust-noise performance.

**Figure 17:**
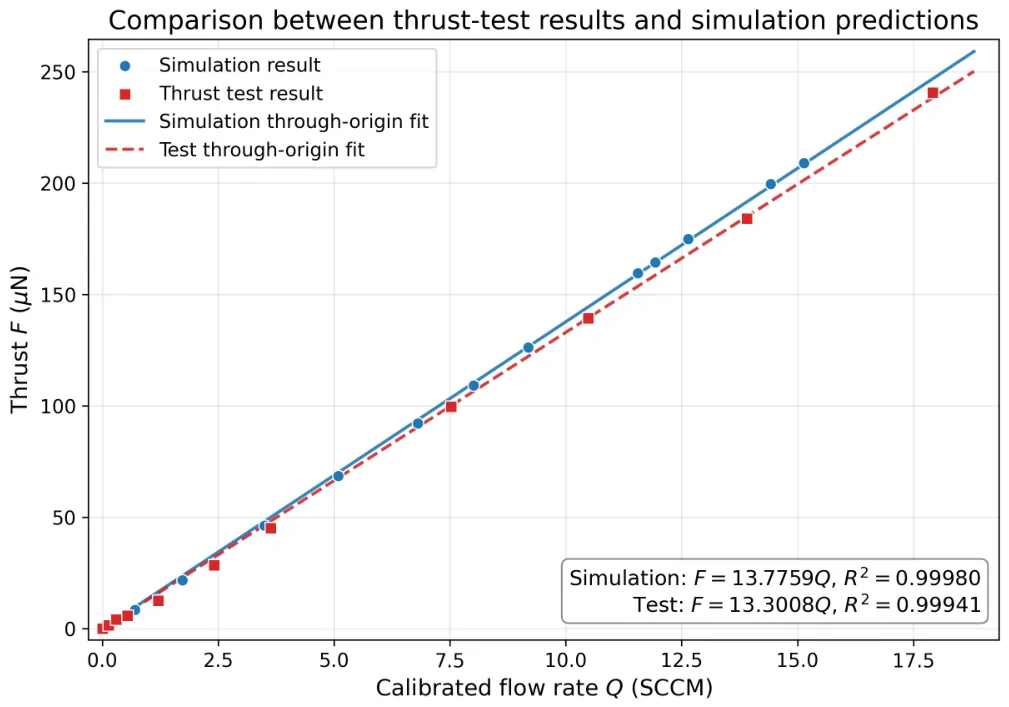
Comparison between the measured flow–thrust relation and the DSMC-based simulation prediction for the fabricated microthruster head, using through-origin linear fits.

Figure 17 compares the thrust-test results with the DSMC prediction using the unified calibrated flow variable *Q* (SCCM). Both datasets exhibit an approximately linear relation between flow rate and thrust over the tested range, and the experimental data follow the same overall trend as the simulation.

A through-origin fit gives F=13.7759Q for the simulation data and F=13.3008Q for the experimental data, with $R^{2}=0.99980$ and $R^{2}=0.99941$, respectively. These results indicate that the measured flow–thrust relation preserves the high linearity predicted by the simulation under the physical constraint of zero thrust at zero flow. The experimental slope is approximately 3.45% lower than the DSMC-fitted slope, indicating that the fabricated device preserves the predicted linearity but produces a modestly lower thrust per unit calibrated flow than the idealized DSMC prediction. This difference may be related to fabrication deviations, assembly misalignment, and additional loss sources in the experimental test configuration.

Overall, the thrust-test results are consistent with the simulation-based flow–thrust trend and support the effectiveness of the integrated thruster-head design. The comparison also shows that the pressure-decay-based calibration provides a consistent basis for thrust evaluation, since the measured thrust data remain quantitatively correlated with the calibrated flow output across the tested range.

Within the nominal target range of 0–200 μN, the measured thrust data remain in good agreement with the DSMC-based flow–thrust trend. The highest tested point extends to approximately 240 μN because a small engineering margin was retained in the PZT stroke design, and this point is therefore treated as a margin-verification result rather than a nominal design-point comparison.

After establishing the flow–thrust correspondence, the measured and simulated specific impulse values were further compared using the same calibrated flow-rate data. For the present work, the calibrated flow variable *Q* is expressed in SCCM and referenced to nitrogen under the standard conditions of $T_{\mathrm{std}}=293.15\;K$ and $P_{\mathrm{std}}=1\;\mathrm{bar}$. The corresponding standard density is

(16) $$\rho_{\mathrm{std}}=\frac{P_{\mathrm{std}}M}{RT_{\mathrm{std}}}\approx 1.1493\;\mathrm{kg}/m^{3},$$

where *M* is the molar mass of nitrogen and *R* is the universal gas constant. The equivalent mass flow is therefore obtained from

(17) $${\dot{m}}_{\mathrm{eq}}=\rho_{\mathrm{std}}\;Q\frac{10^{-6}}{60},$$

which gives ${\dot{m}}_{\mathrm{eq}}\approx 1.9155\times 10^{-8}Q\;\mathrm{kg}/s$ when *Q* is in SCCM. The specific impulse is then calculated from

(18) $$I_{sp}=\frac{F}{{\dot{m}}_{\mathrm{eq}}g_{0}},$$

where $g_{0}$ is the standard gravitational acceleration. Using this relation, the experimental specific impulse was calculated from the measured thrust and calibrated flow rate, while the simulation result was calculated from the corresponding DSMC-based flow–thrust dataset under the same working-gas assumption. Thus, the specific-impulse comparison uses the same calibrated flow definition as the preceding flow–thrust analysis.

**Figure 18:**
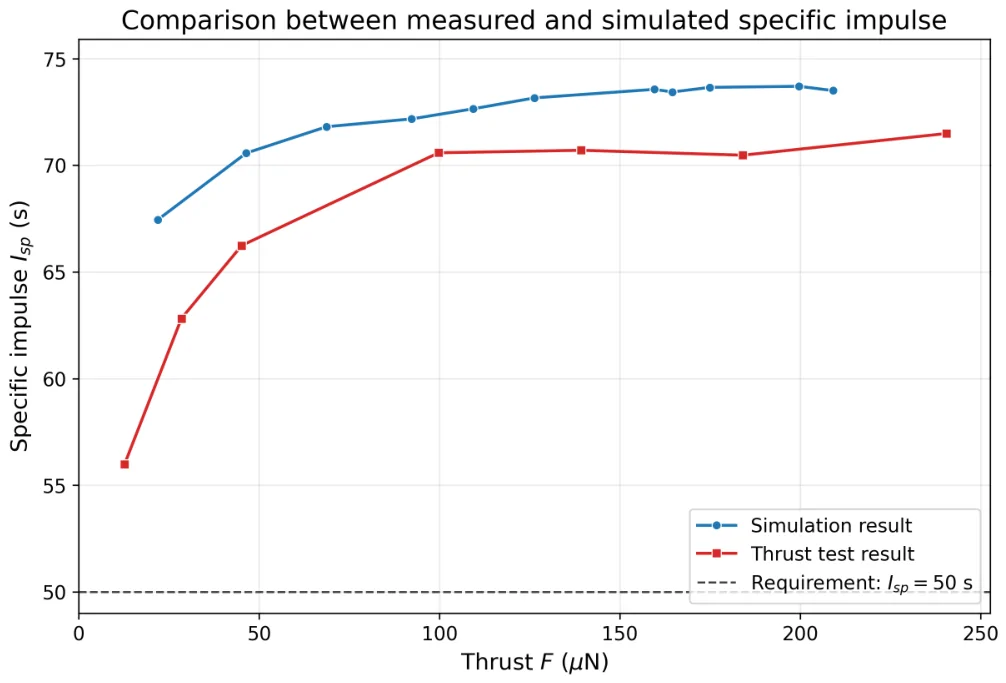
Comparison between the measured and simulated specific impulse values for the fabricated microthruster head. The horizontal dashed line marks the requirement level of $I_{sp}=50\;s$.

Figure 18 shows that both the measured and simulated specific impulse exceed 50 s once the thrust level is above approximately 10 μN, meeting the specified design target defined in Section 1. The measured specific impulse increases from about 56.0 s near the low-thrust end to approximately 71.5 s in the higher-thrust region, while the simulated result remains slightly higher, at about 67.4–73.7 s over the same range. The relative difference is larger near the low-thrust end and becomes smaller as the thrust increases, suggesting that low-flow measurement uncertainty, small throttling-gap deviations, and thrust-stand resolution have a stronger influence in the low-thrust region. This result indicates that the fabricated thruster head preserves the expected nozzle-side momentum-conversion efficiency, although the measured specific impulse is slightly lower than the idealized DSMC prediction.

The thrust-resolution performance was then evaluated under a constant baseline thrust condition. In this test, the thruster was first stabilized at a flow rate of 7.45 SCCM, corresponding to a steady thrust output of approximately 98.4 μN. Small step increases in the commanded flow rate were then applied, with flow increments of 0.004, 0.008, and 0.012 SCCM, while the corresponding thrust response was recorded simultaneously.

**Figure 19:**
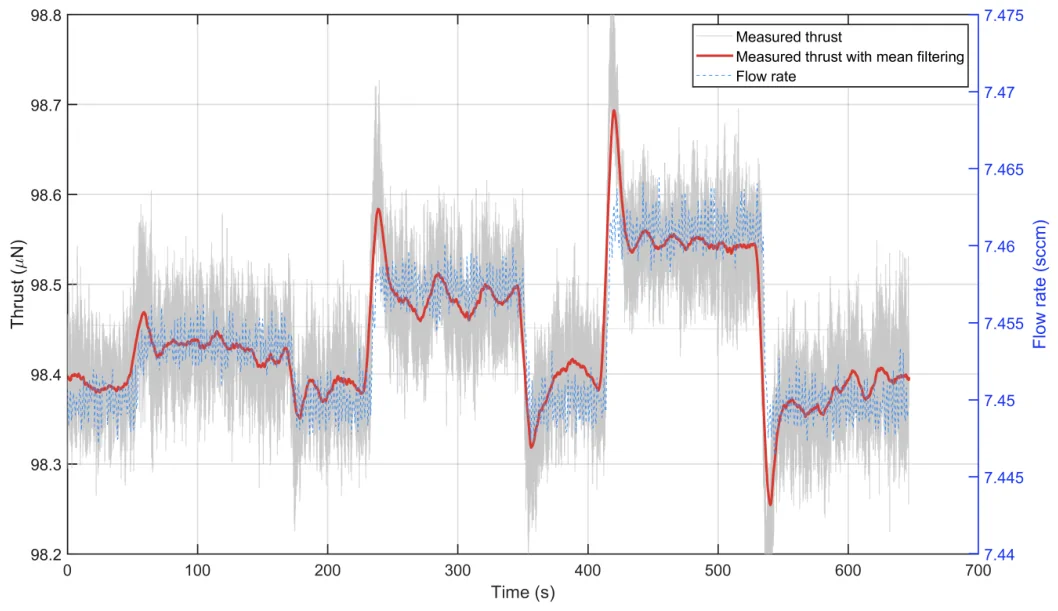
Measured thrust-resolution test at a baseline thrust of approximately 98.4 μN. Step changes in the calibrated flow rate of 0.004, 0.008, and 0.012 SCCM produce clearly distinguishable thrust responses, with the minimum resolved thrust step being approximately 50 nN.

As shown in Figure 19, the smallest applied flow increment of 0.004 SCCM produces a clearly distinguishable thrust step of approximately 50 nN, thereby satisfying the design requirement of 100 nN thrust resolution with margin. In addition, the measured thrust-step amplitudes increase consistently with the imposed flow increments, indicating that the thruster head maintains controllable fine-thrust output under small-signal flow modulation. Accordingly, the reported 50 nN level should be understood as the minimum clearly resolvable thrust step under the present thrust-stand noise floor and calibration conditions.

**Figure 20:**
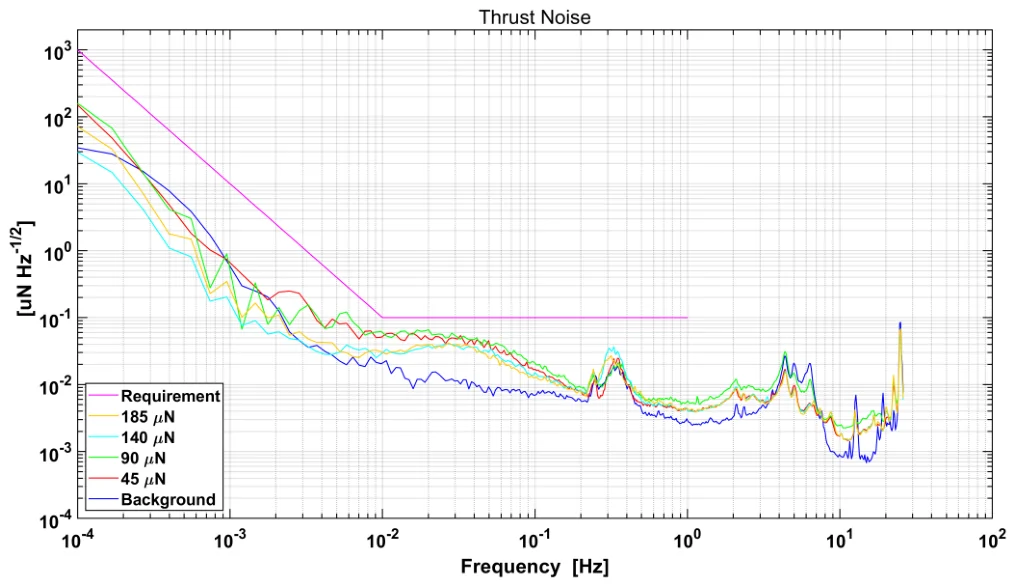
Measured thrust-noise ASD at representative operating levels, compared with the background condition and the requirement curve.

Finally, the thrust-noise results at representative operating levels are shown in Figure 20, including the background condition and the measured thrust levels of approximately 45, 90, 140, and 185 μN. For each operating point, the thrust signal was continuously recorded for approximately 6 h in vacuum and then processed to obtain the amplitude spectral density (ASD), which was used to evaluate the low-frequency thrust-noise behavior. The same processing procedure was applied to all operating levels so that the corresponding spectra could be compared on a consistent basis.

Under the background condition, the pendulum was tested without applying any thrust, so the measured spectrum represents the intrinsic noise floor of the torsional pendulum system. As shown in Figure 20, the background curve exhibits a clear resonance peak in the 0.3–0.4 Hz range, corresponding to the natural frequency of the torsional pendulum, together with another noticeable peak in the 4–7 Hz range that is attributed mainly to environmental disturbances. By comparison, the thrust-noise spectra measured at different thrust levels remain close to each other over most of the analyzed band, indicating that the overall noise level changes only weakly with thrust and that the measured results are consistent across different operating points.

In the 10 mHz–1 Hz band, the measured thrust-noise ASD at all tested thrust levels remains below $0.07\;\mu N/\sqrt{\mathrm{Hz}}$, satisfying the requirement level of $0.1\;\mu N/\sqrt{\mathrm{Hz}}$. For the lower-frequency range of 0.1–10 mHz, the present 6 h record is used only as an indicative low-frequency envelope check. The data provide meaningful coverage down to approximately 1 mHz, whereas the 0.1 mHz point includes only about 2.2 periods and is therefore not sufficient for a statistically stable estimate. Thus, within the 0–200 μN target thrust range, the experimentally supported thrust-noise conclusion is that the measured ASD remains below $0.07\;\mu N/\sqrt{\mathrm{Hz}}$ over the 10 mHz–1 Hz band for the tested thrust levels.

## 6. Discussion

### 6.1. Deviations Between Simulation and Experiment

The experimental results generally agree with the simulation predictions, although measurable deviations remain in the flow–thrust relation. The measured thrust per unit calibrated flow rate is slightly lower than the corresponding DSMC-based prediction. Quantitatively, the experimental flow–thrust slope is about 3.45% lower than the DSMC-fitted value, while the specific-impulse comparison shows a larger difference near the low-thrust end and closer agreement in the higher-thrust region. This result indicates that, although the integrated throttle-nozzle head preserves the predicted linear trend, the fabricated device and test system still introduce non-ideal effects that are not fully captured by the simulation model. In the thrust-noise measurement, the background spectrum is influenced not only by the intrinsic torsional-pendulum response around 0.3–0.4 Hz but also by environmental disturbances in the higher-frequency band around 4–7 Hz.

Several factors may contribute to these discrepancies. First, the fabricated micron-scale geometry may deviate from the nominal design in the throttle interface, throat, and outlet dimensions. Second, assembly alignment errors between the cone needle, valve seat, and downstream nozzle can change the effective throttling condition and nozzle-side momentum conversion. In addition, the thrust-measurement system itself may introduce dynamic errors. Because the target thrust resolution is on the order of 0.1 μN and close to the effective measurement limit of the torsional-pendulum stand, part of the deviation in the low-thrust region may also be associated with the measurement resolution and dynamic response of the pendulum stand. Therefore, the observed differences between simulation and experiment are consistent with the expected combined effects of fabrication, assembly, and measurement-system limitations in the present device-level validation.

### 6.2. Significance of the Integrated Throttle-Nozzle Head

The present results support interpreting the throttle and nozzle as one coupled thruster head rather than as two independent components. The cone-needle throttling interface determines the delivered flow rate and the local upstream condition seen by the nozzle, while the downstream nozzle determines how efficiently this flow is converted into axial thrust. For this reason, the final output characteristic cannot be inferred from valve behavior or nozzle behavior alone.

This coupling also explains why both flow calibration and thrust measurement are required in the validation process. Flow calibration verifies controllable and repeatable gas metering, whereas thrust measurement verifies conversion of the calibrated flow into micro-Newton thrust. Together, these steps provide a coherent device-level validation approach for the proposed integrated cold-gas microthruster head.

### 6.3. Performance Comparison with Representative Micro-Newton Cold-Gas Thrusters

To place the present device-level results in context, Table 6 compares the main performance indicators of the proposed thruster head with representative micro-Newton cold-gas thrusters and mission-oriented requirements. The comparison distinguishes experimentally verified quantities from design-side targets. In particular, the response time of the present head is retained as a design-side constraint, whereas the thrust range, thrust resolution, thrust-noise level, and specific impulse are supported by the ground validation results reported above.

### 6.4. Scope and Limitations of the Present Work

The present work demonstrates a device-level approach for the design, fabrication, calibration, and ground validation of an integrated cold-gas microthruster head, but it should not be interpreted as a flight-qualification campaign. The validation is therefore limited to the fabricated head, the integrated flow-measurement path, and the vacuum ground-test configuration. Dedicated leak-rate and lifetime qualification tests were not included in the present experimental campaign. In addition, because each thrust-noise record lasted approximately 6 h, the noise estimate below about 1 mHz should be regarded as a preliminary low-frequency trend rather than a statistically robust verification, especially at 0.1 mHz. Further tests, including long-duration pressure-decay monitoring, helium leak testing, thermal-vacuum cycling, seal-aging assessment, contamination compatibility checks, and system-level drag-free closed-loop verification, remain outside the scope of this paper. These items will be addressed in the subsequent engineering-level design and qualification stage to quantify the effect of leakage on propellant lifetime and long-term operational stability.

Within this scope, the main contribution of the present study is an experimentally supported validation route that links structural design, rarefied-flow correction, fabrication, calibration, and micro-thrust generation in an integrated cold-gas thruster head.

## 7. Conclusions

This work establishes a device-level design and validation approach for a piezoelectrically controlled cold-gas microthruster head. For a target thrust range of 0–200 μN, one-dimensional theory was first used for preliminary sizing, and DSMC correction of the complete throttle-nozzle geometry was then applied to determine the final design parameters. The selected integrated head uses a throat radius of 29 μm and a needle half-angle of $10^{{}^{\circ}}$, and the fabricated device was subsequently verified through structural characterization, flow calibration, and direct thrust testing.

The experiments show that the pressure-decay-based calibration provides a consistent mapping between actuation command, calibrated flow rate, and thrust output. The fabricated head experimentally supports the nominal 0–200 μN thrust range specified in the initial design, with the highest tested point of about 240 μN treated as a margin-verification result. The thrust-resolution requirement below 0.1 μN is met by the measured 50 nN resolvable step at a baseline thrust of approximately 98.4 μN. The specific-impulse target above 50 s for F≥10 μN is also satisfied over the tested range. For thrust noise, the main experimentally supported result is an ASD below $0.07\;\mu N/\sqrt{\mathrm{Hz}}$ over the 10 mHz–1 Hz band, satisfying the $0.1\;\mu N/\sqrt{\mathrm{Hz}}$ requirement.

The response-time requirement remains a design-side constraint in this work, and statistically robust verification below about 1 mHz, especially at 0.1 mHz, requires longer-duration testing. These results support the proposed integrated cold-gas thruster head as a feasible device-level implementation for stable, controllable, and low-noise micro-thrust generation in future space-based gravitational-wave detection missions.

## Figures and Tables

**Table 1:** Representative state-of-the-art requirements and reported results for micro-Newton thrusters used or proposed for space gravitational-wave missions.

Reference or Platform	Key Indicators	Relevance to the Present Head Design
LISA micropropulsion requirement [14]	Thrust range 2–30 μN; thrust resolution < 0.1 μN; thrust noise < 0.1$\;\mu N/\sqrt{\mathrm{Hz}}$ over the LISA measurement band.	Establishes the science-driven combination of micro-Newton thrust, sub-micro-Newton resolution, and low thrust noise.
LISA Disturbance Reduction System (DRS) thrust-measurement requirement [15]	Continuous thrust 1–100 μN; 0.1 μN resolution; stationary force noise ≤ 0.1$\;\mu N/\sqrt{\mathrm{Hz}}$ from $10^{-4}$ to 1 Hz; dynamic response evaluated up to 10 Hz.	Defines the ground-test capability required to verify range, resolution, noise floor, and response.
LISA Pathfinder cold-gas flight characterization [9]	In-flight cold-gas microthruster operation; average thrust-noise level approximately $0.17\;\mu N/\sqrt{\mathrm{Hz}}$; characterization extended down to about 20 μHz.	Demonstrates flight maturity, while showing that achieving the $0.1\;\mu N/\sqrt{\mathrm{Hz}}$ level remains difficult.
Taiji variable cold-gas prototype [16]	CGMT-50 maximum thrust about 220 μN; 0.05 μN thrust resolution; thrust noise < 0.1$\;\mu N/\sqrt{\mathrm{Hz}}$ from 10 mHz to 1 Hz; thrust response time 140 ms.	Shows recent progress toward wide-range, low-noise, fast-response cold-gas microthrusters and motivates a coupled valve–nozzle–flow-sensing design.

**Table 2:** Target design indicators for the micro-Newton cold-gas thruster head.

Key Indicator	Target	Design Implication
Thrust range	0–200 μN	Sets the required mass-flow range and nozzle momentum conversion.
Thrust resolution	<0.1 μN	Constrains the displacement sensitivity of the throttle interface.
Thrust noise in the science band	<$0.1\;\mu N/\sqrt{\mathrm{Hz}}$ over 10 mHz–1 Hz; low-frequency slope checked against a −40 dB/dec PSD envelope	Couples the noise suppression requirement directly to the mission-sensitive frequency band.
Response time	<150 ms	Constrains dead volume, internal gas-path length, and actuator dynamics.
Specific impulse	>50 s for F≥10 μN	Requires acceptable nozzle expansion efficiency in the mid- and high-thrust range.

**Table 3:** Fixed theoretical baseline geometric parameters used for the subsequent displacement-thrust analysis.

Parameter	Value	Parameter	Value
Needle half-angle θ	$10^{{}^{\circ}}$	Inlet-orifice diameter $D_{i}$	72.36 μm
Stroke range	z=0–50 μm	Throat diameter $D_{t}$	47.07 μm
Exit diameter $D_{e}$	0.235 mm	Diverging-section length $L_{e}$	0.351 mm
Converging-section length $L_{c}$	40.77 μm	Expansion ratio ϵ	25
Expansion half-angle $\alpha_{e}$	$15^{{}^{\circ}}$	Contraction half-angle $\alpha_{c}$	$30^{{}^{\circ}}$
Contraction ratio $\lambda_{c}$	4		

**Table 4:** Representative DSMC candidate comparison used in the second-step displacement-thrust and linearity analysis.

Candidate	Max Thrust (μN)	$R_{F}^{2}$	Interpretation
$R_{t}=25\;\mu m$, $\theta =14^{{}^{\circ}}$	207.80	0.9797	Meets the thrust window with limited linearity margin.
$R_{t}=25\;\mu m$, $\theta =16^{{}^{\circ}}$	218.25	0.9683	Near the upper thrust limit with weaker linearity.
$R_{t}=27\;\mu m$, $\theta =12^{{}^{\circ}}$	201.40	0.9918	Good linearity with a lower thrust ceiling.
$R_{t}=27\;\mu m$, $\theta =14^{{}^{\circ}}$	216.80	0.9848	Meets the thrust window with acceptable linearity.
$R_{t}=28\;\mu m$, $\theta =10^{{}^{\circ}}$	209.51	0.9961	Highest fitted linearity with slightly lower thrust.
$R_{t}=29\;\mu m$, $\theta =10^{{}^{\circ}}$	211.60	0.9957	Selected baseline with high linearity and mid-window thrust.
$R_{t}=30\;\mu m$, $\theta =10^{{}^{\circ}}$	207.80	0.9878	Acceptable thrust with lower linearity.

**Table 5:** Representative DSMC performance of the selected $R_{t}=29\;\mu m$, $\theta =10^{{}^{\circ}}$ design at different throttling displacements.

*z* (μm)	Exit Flow (μg/s)	Thrust (μN)	$I_{\mathrm{sp}}$ (s)	Chamber Pressure (Bar)
1	2.35	1.42	61.51	0.99997
4	14.27	9.28	66.26	0.99996
8	34.02	23.26	69.70	0.99986
16	75.80	54.32	73.09	1.00008
32	180.43	132.53	74.89	1.00011
50	286.00	211.53	75.42	1.00008

**Table 6:** Performance comparison between the present thruster head and representative micro-Newton cold-gas thrusters or mission-oriented requirements.

Reference or Device	Thrust Range	Thrust Resolution	Thrust Noise	Response Time	Specific Impulse	Note
LISA micropropulsion requirement [14]	2–30 μN	<0.1 μN	<$0.1\;\mu N/\sqrt{\mathrm{Hz}}$ over the LISA measurement band	Not specified in the cited requirement summary	Not specified	Mission-driven micropropulsion requirement.
LISA DRS thrust-measurement requirement [15]	1–100 μN	0.1 μN	≤$0.1\;\mu N/\sqrt{\mathrm{Hz}}$ from $10^{-4}$ to 1 Hz	Dynamic response evaluated up to 10 Hz	Not specified	Ground-test capability requirement.
LISA Pathfinder cold-gas flight characterization [9]	In-flight cold-gas microthruster operation	Not specified in the cited characterization summary	Average level about $0.17\;\mu N/\sqrt{\mathrm{Hz}}$	Not specified in the cited characterization summary	Not specified	Flight-demonstrated cold-gas micropropulsion.
Taiji variable cold-gas prototype [16]	Maximum thrust about 220 μN	0.05 μN	<$0.1\;\mu N/\sqrt{\mathrm{Hz}}$ from 10 mHz to 1 Hz	140 ms	Not specified in the cited summary	Recent wide-range low-noise prototype.
Present work	0–200 μN nominal; tested to about 240 μN as margin	50 nN resolvable step at about 98.4 μN	<$0.07\;\mu N/\sqrt{\mathrm{Hz}}$ over 10 mHz–1 Hz	<150 ms design-side target	56.0–71.5 s measured; >50 s for F≥10 μN	Device-level ground validation of the integrated head.

## Data Availability

The data and scripts supporting the findings of this study are available from the corresponding author upon reasonable request.

## Abbreviations

The following abbreviations are used in this manuscript:

ASD	Amplitude spectral density
DSMC	Direct Simulation Monte Carlo
MEMS	Micro-electro-mechanical systems
PCB	Printed circuit board
PEEK	Polyether ether ketone
PSD	Power spectral density
PZT	Lead zirconate titanate piezoelectric actuator
SCCM	Standard cubic centimeters per minute
